# Triage Strategies for Critically Ill Patients With Acute Myocardial Infarction Requiring Invasive Mechanical Ventilation

**DOI:** 10.1016/j.jacadv.2024.101197

**Published:** 2024-08-15

**Authors:** Shashank S. Sinha, Patrick R. Lawler

**Affiliations:** a Inova Schar Heart and Vascular, Inova Fairfax Medical Campus, Falls Church, Virginia, USA; b Division of Cardiology, McGill University Health Centre, Montreal, Quebec, Canada; c Division of Cardiology and Interdepartmental Division of Critical Care Medicine, University of Toronto, Toronto, Quebec, Canada

**Keywords:** acute myocardial infarction, intensive care unit, cardiogenic shock

**Figure:**
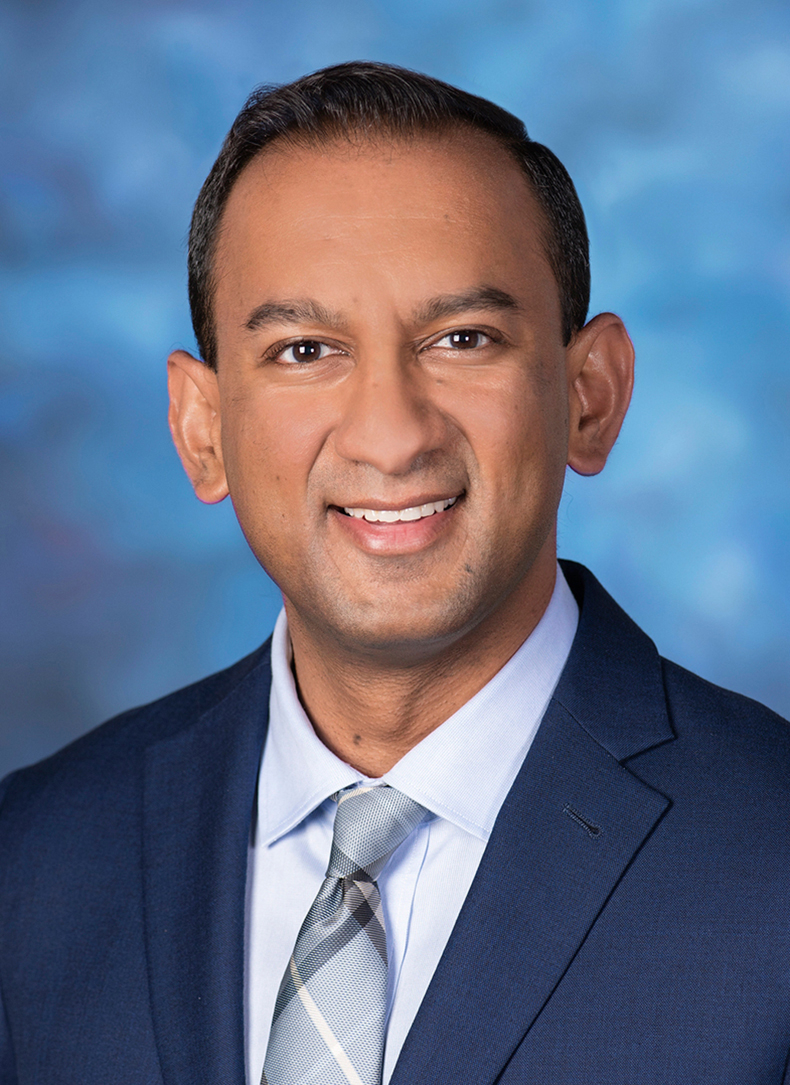

Cardiac intensive care units (CICUs) have evolved over the past several decades to manage critically ill patients with cardiovascular disease across a broad array of both cardiovascular and noncardiovascular indications.^1^, ^2^, ^3^ Respiratory failure has now emerged as a common condition at the time of admission to contemporary CICUs at tertiary and quaternary care centers,^3^ including for patients with acute myocardial infarction (AMI).^4^ While hospitals and health care systems may have local or other specific factors that determine whether critically ill patients with AMI are admitted to a CICU vs a more general medical intensive care unit (MICU), there is a paucity of evidence on the clinical characteristics, resource utilization, and in-hospital outcomes for patients with AMI admitted to each of these settings.

In this issue of *JACC: Advances*, Drs Shadu, Miller, and colleagues present the results of a cohort study to compare characteristics and outcomes among patients with AMI and concomitant respiratory failure admitted to a CICU vs MICU.^5^ The investigators analyzed >12,000 patients with AMI requiring invasive mechanical ventilation (IMV) admitted to either a MICU (n = 3,185) or CICU (n = 9,454) using the Vizient Clinical Database (which encompasses >95% of academic medical centers and >300 community hospitals in the United States) from October 2015 to December 2019. Only patients admitted to a MICU or CICU were included in this study. The primary outcome was in-hospital mortality. Secondary outcomes included discharge status and disposition, total hospital cost, number of days on the ventilator, and hospital and intensive care unit (ICU) length of stay.

Patients with AMI requiring IMV admitted to a CICU, as compared to those admitted to an MICU, were more likely to be male (67.6% vs 64.5%, *P* = 0.001), more likely to be admitted to a teaching hospital and/or large hospital (ie, >750 beds) (*P* < 0.001 for both), and more likely to present with ST-segment elevation myocardial infarction (STEMI) (57.0% vs 42.8%, *P* < 0.001) or cardiogenic shock (46.0% vs 37.4%, *P* < 0.001).^5^ Conversely, patients admitted to the MICU were more likely to have non-STEMI, cardiac arrest, and renal failure on admission (all *P* < 0.001).^5^ Perhaps not surprisingly, patients admitted to the CICU were more likely to have cardiovascular comorbidities, including pre-existing coronary artery disease, prior myocardial infarction, prior revascularization, and history of heart failure.^5^

The investigators were unable to establish the etiology of respiratory failure—a key limitation of this analysis. Notably, there were no significant differences between ICU types for other noncardiac procedures and therapies, including renal replacement therapy, blood transfusions, and tracheostomy. Furthermore, utilization of critical care therapies was similar when the authors performed a sensitivity analysis stratified by STEMI vs non-STEMI as the primary diagnosis.

The unadjusted in-hospital mortality of AMI patients requiring IMV was not statistically different between those admitted to a CICU as compared to a MICU (41.3% vs 42.7%, *P* = 0.15).^5^ However, after multivariable adjustment for demographics, comorbidities, hospital characteristics, and illness severity, CICU admission was associated with lower in-hospital mortality (OR: 0.85; 95% CI: 0.78-0.93; *P* < 0.001).^5^ This association persisted when patients were stratified by cardiogenic shock, cardiac arrest, STEMI, largest hospital size, and teaching hospitals (all *P* < 0.05).^5^

This analysis contributes to a growing literature related to triage practices and outcomes among critically ill patients with acute cardiovascular diseases.^6^^,^^7^ Evidence-based criteria to reduce clinical variation in CICU utilization and overall triage practices are largely lacking. Although CICU-appropriate use criteria have been proposed to guide triage and admission,^8^ no large, multicenter, prospective randomized controlled trials have formally or rigorously tested triage-based algorithms to the CICU. There is an unmet opportunity for such trials, which may employ registry-based, cluster-randomized, or stepped-wedge trial designs.^9^

Key strengths of this observational retrospective analysis were that the clinical database enabled direct comparison of patients in CICUs with those in MICUs specifically, as opposed to any “noncardiac” ICU (eg, a cardiac surgical ICU, neurological ICU, etc). Moreover, only patients with complete data were included, and missingness was extremely rare in this database. Limitations of this analysis include the lack of data on ICU organizational structure, staffing models (“high” vs “low” intensity staffing, physician certification, etc), and hospital-level data regarding institutional levels of care (eg, presence or absence of intermediate care units and their capabilities). These local factors would be anticipated to have a large impact on optimal triage at the hospital level. Thus, it may be difficult to draw institutionally-relevant conclusions from large observational studies such as this one.

Observational data also suggests that the presence of a critical care specialist, either in the form of a critical care cardiologist or a co-management model between a cardiologist and intensivist, has been associated with improved clinical outcomes, especially among patients with respiratory failure.^10^^,^^11^ This remains an important data gap: Which patient should be triaged to a cardiac ICU? The local institutional solution likely reflects an amalgam of factors including but not limited to physician knowledge and procedural skills, nursing structure and expertise, allied health professional support, hospital levels of care, and many others.

In summary, should patients with AMI requiring IMV be triaged and admitted to the CICU or MICU? The answer remains complicated and very likely depends on a range of patient-specific and hospital-level factors. Nevertheless, on a broad level, the current data suggest that outcomes are broadly similar when patients with respiratory failure complicating AMI are managed in the CICU, suggesting that such patients may, in many cases, be well cared for in the contemporary CICU.

## Funding support and author disclosures

The authors have reported that they have no relationships relevant to the contents of this paper to disclose.
